# Evaluation of Fever of Unknown Origin in an Infant: Can a Case Be Made for Ultrasound of the Head as a Second-Tier Test for Meningitis?

**DOI:** 10.7759/cureus.96850

**Published:** 2025-11-14

**Authors:** Aaron F Osborne, Mariah A Jordan, Olivia Perdigon, Angelica N Byrd, Mobeen Rathore

**Affiliations:** 1 Pediatric Infectious Diseases, University of Florida College of Medicine – Jacksonville, Jacksonville, USA; 2 Pediatrics, University of Florida College of Medicine – Jacksonville, Jacksonville, USA

**Keywords:** antibiotic administration, fever of unkown origin, head ultrasound, high fever, meningitis, pediatric infectious diseases, pediatrics

## Abstract

Although meningitis in neonates has been less prevalent since the initiation of prevention measures such as maternal group B streptococcal (GBS) screening and intrapartum antibiotic prophylaxis, it remains on the differential diagnosis for causes of prolonged fever or fever of unknown origin (FUO). Although not always present, fever is a potential symptom of meningitis in neonates and infants, in whom other signs and symptoms of meningitis are not as easily identifiable as in older children. Cerebrospinal fluid (CSF) analysis and culture remain the gold standard for diagnosis of meningitis, but ancillary testing, such as head imaging, can aid in the evaluation of the patient. We present a case of a two-month-old male child in which a head ultrasound via the anterior fontanelle was able to aid in the diagnosis of meningitis.

## Introduction

Although there is no universally accepted definition of fever of unknown origin (FUO), most physicians accept a fever of greater than 100.4° F for more than eight days as an FUO. The evaluation of FUO is as much art as it is science, and infection is the most common cause of FUO [1].

Meningitis is inflammation of the leptomeninges. Viral infection is the most common etiology of meningitis in children of all ages. Although its clinical presentation is often non-specific, especially in infants, symptoms may include temperature instability (hyperthermia or hypothermia), irritability, lethargy, seizures, and decreased feeding. Although not always present, fever is one of the major presentations of meningitis.

Along with meningitis caused by herpes simplex virus, bacterial pathogens are the most common treatable causes of meningitis. *Escherichia coli* and GBS are the most prevalent causes of neonatal meningitis. Rates of neonatal bacterial meningitis have plummeted since the initiation of maternal group B streptococcal (GBS) screening and intrapartum antibiotic prophylaxis, occurring in only 1% of neonates [2]. In non-neonatal infants, especially those who are unimmunized,* Streptococcus pneumoniae* and rarely *Hemophilus influenzae* type b are the most common causes of bacterial meningitis, as is *Neisseria meningitidis* [3,4]. 

Broad-spectrum antibiotics should be started as soon as possible if meningitis is suspected. Obtaining a cerebrospinal fluid (CSF) specimen to determine the pathogenic cause of meningitis is preferred and highly recommended before initiating antibiotic therapy. However, antibiotic initiation should not be delayed due to the effort to obtain a CSF specimen. Selection and duration of antimicrobial therapy are age and pathogen-dependent and should be tailored to the identified pathogens and their susceptibilities [5]. In cases of pre-treatment, CSF may not yield a positive culture, and in such cases, a molecular test can be valuable in pathogen identification. Even without a pathogen identified, other CSF findings can diagnose meningitis. A CSF specimen with an elevated white blood cell (WBC) count, low glucose, and elevated protein is strongly suggestive of meningitis [6]. Gram stain, culture, and molecular testing on the CSF specimen may identify the pathogen causing meningitis. Magnetic resonance imaging (MRI) is the optimum imaging modality, but not essential for the diagnosis of meningitis [7]. Findings of meningeal thickening and hyperemia are suggestive of bacterial meningitis, but ultrasound is not usually recommended in the evaluation of meningitis, as it is not considered sensitive or specific [8]. However, recent investigations have shown the potential of high-resolution head ultrasound used in conjunction with multi-stage deep learning models to screen for meningitis in infants [9].

We report a case that was originally classified as a case of FUO but later diagnosed as meningitis, likely bacterial, with the aid of abnormal head ultrasound findings.

## Case presentation

A two-month-old previously healthy male infant born to a GBS-negative mother presented with a 10-day history of fever. His laboratory evaluation at this time was notable for a WBC count of 26.71 × 10^9^/L with 57.5% segmented neutrophils, 30.6% lymphocytes, 10.8% monocytes, 0.3% eosinophils, and 0.8% basophils, platelet count of 723 x 10^9^/L, C-reactive protein (CRP) of 220 mg/L (reference range, 5-10 mg/L), erythrocyte sedimentation rate (ESR) of 29 mm/hour, two sterile peripheral blood cultures, a sterile urine culture, normal abdominal ultrasound, and unremarkable echocardiogram. He was treated with one dose of intravenous (IV) ceftriaxone. Fever resolved 72 hours later, and he was discharged on hospital day 5 after remaining afebrile for more than 48 hours.

Five days after discharge, the infant presented again with a one-day history of fever. The patient's repeated episodes of fever were partially relieved by acetaminophen and had associated symptoms of significantly decreased oral intake, two episodes of non-bloody, non-bilious vomiting, and increased fussiness while febrile. His bowel movements and urine output were appropriate in amount and frequency. There was no history of cough, rash, edema, hematuria, abnormal movements, or known sick contacts.

On examination, the patient had a rectal temperature of 101°F, heart rate of 168/minute, respiratory rate of 56/minute, blood pressure of 103/76 mm Hg, and oxygen saturation of 98% in room air. Physical examination was notable for generalized pallor, mild congestion, and slight erythema of non-bulging tympanic membranes but without evidence of middle-ear effusion, bilaterally. Laboratory evaluation was significant for leukocytosis with a WBC count of 56.70 × 10^9^/L with 87.4% segmented neutrophils, 7.9% lymphocytes, 4% monocytes, and 0.7% basophils, platelet count of 1593 x 10^9^/L, and CRP of 130 mg/L. Comprehensive metabolic panel and urinalysis were unremarkable. The molecular respiratory pathogen panel was negative. Repeat blood culture was obtained and remained sterile. A repeat echocardiogram and an abdominal ultrasound were unremarkable.

**Figure 1:**
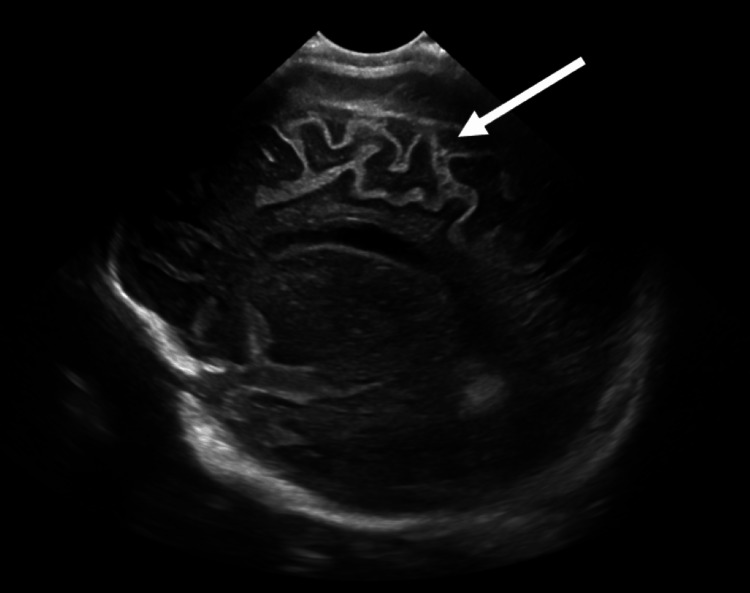
Head ultrasound, right sagittal view, demonstrates widened and echogenic cerebral sulci (arrow).

**Figure 2:**
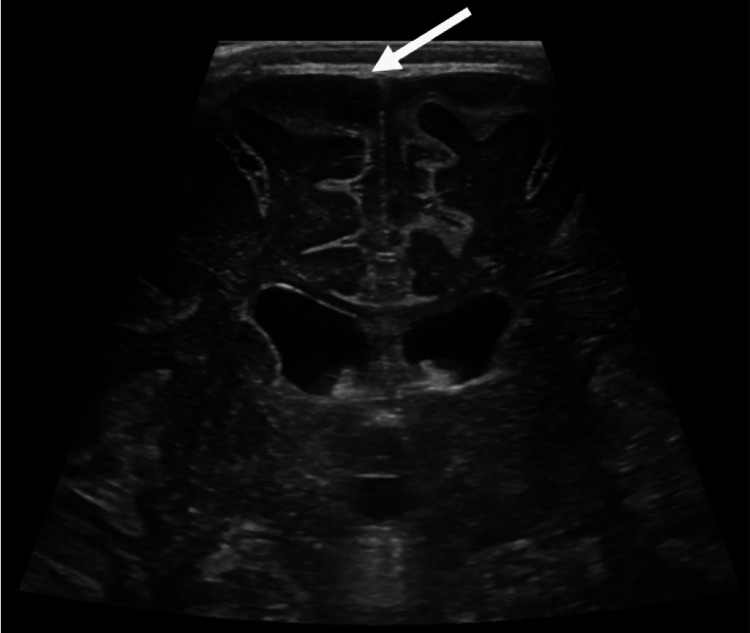
Head ultrasound, coronal mid view, demonstrates a prominent extra-axial fluid space (arrow).

The patient was admitted to undergo further evaluation. Continued fevers and fussiness led to obtaining a head ultrasound that revealed widened and echogenic cerebral sulci and prominent extra-axial fluid spaces suggestive of meningitis (Figure 1, Figure 2). A CSF specimen showed 1478 WBC/μL with 97% neutrophils, 2% lymphocytes, and 1% monocytes, glucose of 11 mg/dL, and protein of 176 mg/dL. These values were also indicative of meningitis. Empiric IV ceftriaxone and vancomycin were started.

**Figure 3:**
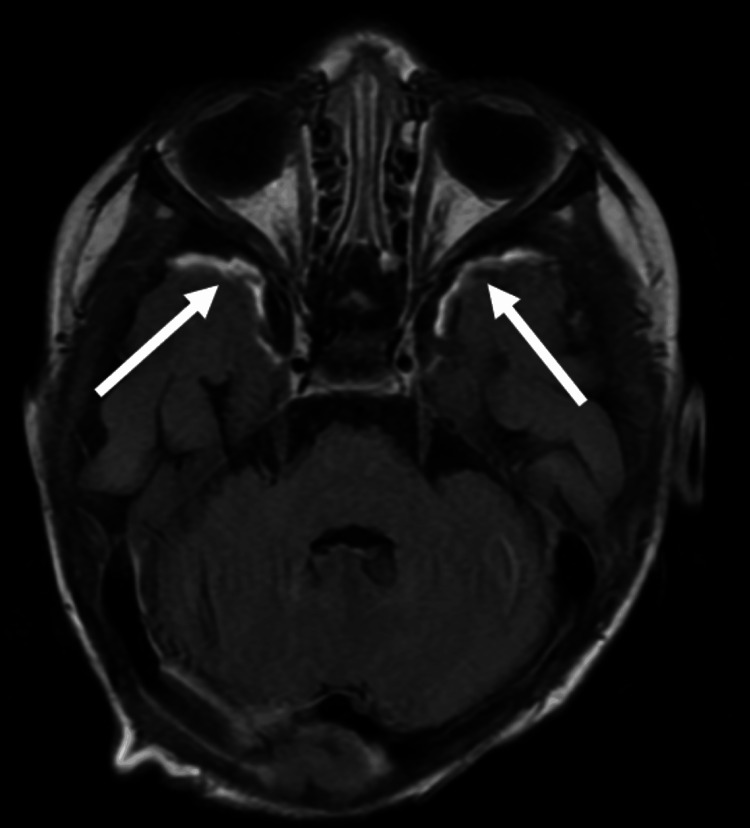
MRI brain, axial view, FLAIR T2 post-contrast, shows the meningeal enhancement in the floor of the anterior cranial fossa bilaterally (arrows). FLAIR: fluid-attenuated inversion recovery

MRI brain with contrast enhancement obtained two days later showed evidence of ventriculitis and purulent material in the bilateral and interhemispheric subdural space with surrounding meningeal enhancement consistent with a subdural empyema (Figure 3). The CSF Gram stain, meningitis/encephalitis polymerase chain reaction (PCR) panel (BIOFIRE® FILMARRAY® Meningitis/Encephalitis Panel; bioMérieux SA, Marcy-l'Étoile, France), and 16S ribosomal RNA (rRNA) gene PCR were negative, and CSF culture remained sterile.

The neurosurgeon recommended conservative therapy for the subdural empyema, and the patient was treated with four weeks of intravenous antibiotics. A repeat MRI was obtained prior to the end of four weeks of antibiotic therapy and showed an interval decrease in the size of the subdural empyema and resolution of the signs of meningitis.

Imaging findings and impressions are summarized in Table 1. The patient improved clinically, his CRP and platelet levels became normal, and he was discharged home. Six days after stopping antibiotics, he was doing well without any sequelae.

**Table 1:** Summary of imaging findings and impressions

Imaging Modality	Pertinent Findings	Impression
Neonatal Head Ultrasound	Increased echogenicity of the extra-axial spaces, thickened meninges and sulcal widening, lateral and third ventricles are mildly prominent, vasodilatation of prominent vascularity along the surface of the brain, and no obvious intraventricular debris or ependymal increased echogenicity.	Sonographic findings highly concerning for meningitis. Mildly prominent lateral and third ventricles.
MRI Brain with and without Contrast – Pretreatment	Restricted diffusion in the right occipital horn suggestive of ventriculitis, bilateral subdural space along the convexity as well as in the interhemispheric subdural space show few foci of restricted diffusion and meningeal enhancement suggestive of presence of pus in this region, meningeal enhancement in bilateral middle cranial fossa as well as in the floor of the anterior cranial fossa bilaterally, ventricles and sulci are prominent, and no prominent extra-axial fluid in the posterior fossa.	Meningitis. Ventriculitis. There is presence of small amount of pus in bilateral subdural fluid with surrounding meningeal enhancement consistent with developing subdural empyema.
MRI Brain with and without Contrast – Post treatment	Again sulci are prominent, tiny foci of restricted diffusion along the interhemispheric falx significantly decreased in comparison to prior study, significant interval improvement of the previously seen meningeal enhancement, ventricles normal in configuration and again prominent in size, and interval improvement of the previously seen restricted diffusion within the occipital horn of the right lateral ventricle.	Significant interval decrease in size subdural empyema and meningitis seen on prior study, with only trace residual foci of restricted diffusion and meningeal enhancement visualized. Interval resolution of the intraventricular restricted diffusion related to previous ventriculitis. No significant new interval abnormalities are visualized.

## Discussion

A combination of the patient’s clinical picture and imaging findings on head ultrasound suggested meningitis. CSF analysis with pleocytosis with neutrophilic predominance, hyperproteinorrachia, and hypoglycorrhachia confirmed meningitis, which was supported by the imaging findings. Bacterial meningitis, complicated by ventriculitis and subdural empyema, was highly likely. Meningococcal or pneumococcal meningitis is high on the differential in this patient, as some data suggest that a single dose of a third-generation cephalosporin, like the ceftriaxone the patient in our case received, may be sufficient to sterilize the CSF [10]. Meningococcal disease can have high morbidity and mortality. However, early antibiotic treatment, presence of only mild symptoms, lack of neurological symptoms, absence of septicemia and shock, lack of skin findings, and absence of thrombocytopenia and neutropenia are factors associated with better outcomes. Our patient met all these criteria [11].

Evaluation of FUO requires a thoughtful, structured, and tiered approach, starting with the least invasive testing and focusing on the most common and treatable conditions [1]. When evaluating prolonged fevers, it is important to consider urinary tract infections, acute otitis media, pneumonia, bacteremia, meningitis, and immune-related processes like Kawasaki Disease in the differential diagnosis. All of which were examined in this patient. Urinary tract infection and acute otitis media were ruled out. Occult bacteremia or a partially treated bacteremia is unlikely since blood cultures pre- and post-one dose of ceftriaxone remained sterile. The patient did not meet the criteria for Kawasaki Disease [12]. A viral infection not included on the respiratory pathogen panel was the most likely diagnosis during the first admission. Upon readmission, malignancy and hemophagocytic lymphohistiocytosis were ruled out.

A high index of suspicion for meningitis is needed in febrile infants, even when blood and urine cultures are sterile and the patient is well appearing between febrile episodes, as a delay in treatment can result in high morbidity and mortality [3].

CSF analysis and culture remain the gold standard for the diagnosis of meningitis. As the patient appeared well, a lumbar puncture had been deferred during the previous admission. On readmission, the persistence of the fevers and abnormal head ultrasound findings ultimately tipped the scales and led to the lumbar puncture and ultimately the diagnosis of meningitis. The success rate of lumbar punctures in infants less than three months of age is 69% [13], and it is important that pediatric providers utilize other available diagnostic information to aid in clinical decision-making.

Although limited by small sample size and the everchanging landscape of deep learning, 100% sensitivity and 90% specificity for high resolution head ultrasound detection were cited in a recent, 16-patient study where an AI model was trained in a three-stage process including quality control, screening to distinguish between control images and images with patterns indicative of WBCs in CSF, and explainability analysis [9]. While there was no AI or deep learning used in this patient’s case because the head ultrasound was obtained due to clinical suspicion, there is promise for this noninvasive and cost-effective imaging modality to help screen for meningitis in infants; however, it warrants additional study.

## Conclusions

This case report highlights the importance of continued evaluation of prolonged fevers in pediatric patients and the utility of both noninvasive and invasive testing. In the evaluation of FUO, a tiered approach in which the provider can tailor testing according to the patient’s presentation is warranted to identify the cause of fever while avoiding costly and potentially harmful procedures. While head ultrasound is not as specific or sensitive for meningitis as an MRI, it requires further research as a possible second-tier test in the evaluation of FUO in infants, as ultrasound has no radiation exposure, is noninvasive, cost-effective, widely available, does not require sedation, and may demonstrate findings of meningitis that warrant further investigation.
